# Methodological Concerns Regarding the Bayesian Network Meta-Analysis of Telerehabilitation for Chronic Low Back Pain

**DOI:** 10.2196/106237

**Published:** 2026-09-16

**Authors:** Adili Tuersun, Guo Ma

**Affiliations:** 1 School of Pharmaceutical Sciences, State Key Laboratory of Advanced Drug Formulations for Overcoming Delivery Barriers, Fudan University, 825 Zhangheng Road, Pudong New District, Shanghai, 201203, China, 86 17624142443

**Keywords:** Bayesian network meta-analysis, artificial intelligence, chronic nonspecific low back pain, in-person rehabilitation, telerehabilitation, usual care

We read the recent Bayesian network meta-analysis (NMA) by Gu et al [1] comparing telerehabilitation combined with AI (TLRH-AI), telerehabilitation, in-person rehabilitation, and usual care for chronic nonspecific low back pain. The study raises an important clinical question, yet methodological shortcomings compromise the validity and interpretability of the findings.

For the risk of bias assessment, the authors describe using the Cochrane Risk of Bias (RoB) tool (2.0), as implemented in Review Manager (RevMan; version 5.4.1). This is problematic because RevMan 5.4.1 lacks support for RoB 2.0, and their study-level 7-domain assessment confirms that RoB 1.0 was in fact applied [2]. Such misrepresentation risks misleading readers about the rigor of the bias assessment.

The authors also used *I*^2^ to describe statistical heterogeneity within a Bayesian framework, treating values above 50% as indicative of substantial heterogeneity. This is inappropriate. Within a Bayesian NMA, heterogeneity is quantified through τ, whose posterior distribution should be reported [3]. Although the authors mention specifying τ ∼ Uniform (0, om.scale), they do not provide posterior medians or credible intervals across comparisons. Relying on *I*^2^, a metric with recognized frequentist limitations in NMA, masks the extent of between-study variance and makes it difficult to judge the robustness of network estimates.

Marked baseline imbalances are apparent in Supplementary Table 8 in Multimedia Appendix 1 of Gu et al [1]. In the TLRH-AI group, the mean age was 33.2 (range 29.3-37.1) years, whereas the telerehabilitation, in-person rehabilitation, and usual care groups had mean ages of 43.0, 45.4, and 46.2 years, respectively. Pain and disability severity at baseline also differed. Age and baseline severity are established prognostic factors for recovery in chronic low back pain. The authors’ claim that characteristics were “broadly comparable” finds little support in the data, and combining these populations without sensitivity analyses or meta-regression calls into question the transitivity assumption that underpins NMA [3]. Should transitivity be violated, the indirect comparisons that support the network estimates, and consequently the surface under the cumulative ranking curve values reported in the abstract, become unreliable. These risk generating inappropriate recommendations that favor AI-assisted telerehabilitation for older patients or those more severely affected, groups that were not well represented in the TLRH-AI trials.

Markov chain Monte Carlo convergence was assessed through “visual inspection” without formal reporting of R̂ values or effective sample sizes [4]. In Bayesian analysis, these diagnostics are essential to confirm that posterior distributions have been adequately sampled and that credible intervals are trustworthy. Without them, readers cannot verify the reliability of network estimates. The authors also inserted fixed τ values to calculate 95% prediction intervals when posterior estimates were unavailable. This is problematic because prediction intervals should derive from the model’s posterior distribution of τ [5]. Manually inserting τ values departs from the Bayesian paradigm and produces intervals that are not model-based and may not be valid for predicting effects in future studies.

We invite the authors to address these methodological and statistical concerns, explaining their analytical choices and supplying the missing diagnostics and posterior estimates. Clarifying these issues would help readers and clinicians judge the robustness of the conclusions before the findings inform clinical practice or policy.

## Abbreviations

NMA: network meta-analysis
RevMan: Review Manager
RoB: Cochrane Risk of Bias
TLRH-AI: telerehabilitation combined with AI
